# Tangshen formula attenuates diabetic renal injuries by upregulating autophagy via inhibition of PLZF expression

**DOI:** 10.1371/journal.pone.0171475

**Published:** 2017-02-09

**Authors:** Hailing Zhao, Xin Li, Tingting Zhao, Haojun Zhang, Meihua Yan, Xi Dong, Pengmin Chen, Liang Ma, Ping Li

**Affiliations:** 1 Beijing Key Lab Immune-Mediated Inflammatory Diseases, Institute of Clinical Medical Sciences, China–Japan Friendship Hospital, Beijing, China; 2 Clinical laboratory, China–Japan Friendship Hospital, Beijing, China; University of Utah School of Medicine, UNITED STATES

## Abstract

The Chinese herbal granule Tangshen Formula (TSF) has been proven to decrease proteinuria and improve estimated glomerular filtration rate (eGFR) in diabetic kidney disease (DKD) patients. However, the underlying mechanism of TSF on treatment of diabetic nephropathy (DN) remains unclear. The present study aimed to identify the therapeutic target of TSF in diabetic renal injuries through microarray-based gene expression profiling and establish its underlying mechanism. TSF treatment significantly attenuated diabetic renal injuries by inhibiting urinary excretion of albumin and renal histological injuries in diabetic (db/db) mice. We found that PLZF might be the molecular target of TSF in DN. *In vivo*, the db/db mice showed a significant increase in renal protein expression of PLZF and collagen III, and decrease in renal autophagy levels (downregulated LC3 II and upregulated p62/SQSTM1) compared to db/m mice. The application of TSF resulted in the downregulation of PLZF and collagen III and upregulation of autophagy level in the kidneys of db/db mice. *In vitro*, TSF reduced high glucose (HG)-induced cell proliferation for NRK52E cells. Further studies indicated that the exposure of NRK52E cells to high levels of glucose resulted in the downregulation of cellular autophagy and upregulation of collagen III protein, which was reversed by TSF treatment by decreasing PLZF expression. In conclusion, TSF might have induced cellular autophagy by inhibiting PLZF expression, which in turn resulted in an increase in autophagic degradation of collagen III that attenuated diabetic renal injuries.

## Introduction

Diabetic nephropathy (DN) was one of the major microvascular complications of type 2 diabetes mellitus (T2DM) and was also a major cause of end-stage renal disease (ESRD). Key pathological features of DN included glomerular hypertrophy, as well as extracellular matrix (ECM) deposition in the mesangium and tubulointerstitium [1]. Clinically, microalbuminuria was considered to be an important marker of DN, which reflected not only glomerular injuries but also tubular lesions [2]. The present study showed that the promyelocytic leukemia zinc finger protein (PLZF) might be a pharmacologic target of DN. PLZF, encoded by zinc finger BTB domain containing 16 (ZBTB16), belonged to the BTB/POZ-ZF protein family [3]. It was a transcription factor that had been found to regulate a wide variety of biological processes including cell proliferation, differentiation and apoptosis [4, 5]. However, there was not enough researches on PLZF in the pathogenesis of DN.

Autophagy was a highly conserved and lysosome-dependent bulk degradation pathway that participated in the clearance of damaged organelles and proteins, as well as maintained homeostasis in tubules and glomeruli [6]. Previous studies have implicated deficient autophagic activity in the pathogenesis of diabetic kidney injuries [7–9]. Microtubule-associated protein 1 light-chain 3 (LC3) was often employed in monitoring the cellular autophagy. The level of LC3 II has also been used as a marker protein for the determination of activity and progression of autophagy. p62/SQSTM1, an important substrate for autophagy degradation, was another common marker protein that had been utilized in the determination of autophagy activity. Unlike LC3 II, the expression level of p62/SQSTM1 decreased during autophagy progression, whereas it increased with the inhibition of autophagy [10]. Recent studies have involved autophagy in the development of effective therapeutic strategies for DN [7, 11].

Chinese herbal medicine (CHM) has been widely used in the treatment of diabetes and its complications including DN in China [12, 13]. Tangshen Formula (TSF) was a CHM remedy for diabetic renal injuries. In our previous studies, we have demonstrated that TSF was significantly efficacious in alleviating proteinuria and improving estimated GFR (eGFR) in diabetic kidney disease (DKD) patients [14]. TSF had therapeutic potential for type 2 DN in rats through blockade of NF-κB-driven renal inflammation and TGF-β/Smad3-mediated renal fibrosis [15]. Nevertheless, little was known on the underlying mechanisms of TSF on treating DN. In the present study, we explored the target genes of TSF in the treatment of DN as well as investigated the molecular mechanism of the target gene in relation to the development of DN.

## Materials and methods

### Herbal formulation and components

TSF consisted of 7 natural herbs: 35.3% astragalus root (Astragali radix), 17.6% burning bush twig (Euonymi ramulus), 14.4% rehmannia root (Rehmanniae radix), 11.5% bitter orange (Aurantii fructus), 10.6% cornus fruit (Corni fructus), 7.1% rhubarb root and rhizome (Rhei radix et rhizoma), and 3.5% notoginseng root (Notoginseng radix). We prepared and standardized TSF powder in an established company (Jiangyin Tianjiang Pharmaceutical, Jiangsu, China, http://www.tianjiang.com) using the high-quality control standards of China. The preparation of the herbal drugs was performed according to the established guidelines in the Pharmacopoeia of The People’s Republic of China 2010.

In our previous research, TSF had been subjected to high performance liquid chromatography (HPLC) analysis, and its chemical fingerprint was identified at 254 nm, including loganin, calycosin-7-O-β-D-glucoside, naringenine-7-rhamnosidoglucoside, neohesperidin, naringenin and Aloe-emodin [16].

### Animals and experimental design

Mice were purchased from the Peking University Laboratory Animal Center (Beijing, China), including 8-week-old male C57BLKS/J db/db (n = 18) and db/m mice (n = 9), and db/m mice were used as controls. Mice were housed using controlled humidity (55 ± 15%) and temperature (23 ± 3°C), with a 12-h light-dark cycle. The mice were allowed access to standard laboratory food and water ad libitum. The db/db mice were divided into 2 groups (n = 9 for each group): one was given TSF by intra-gastric gavage (2.4 g/kg/day), whereas the other was administered saline. After 12 weeks of TSF treatment, blood and tissues were collected for further analysis. According to a previous clinical study and standard conversion formula, the effective dose of TSF in mice is equivalent to 2.4 g/kg/day [14]. The Ethics Committee of the China-Japan Friendship Institute of Clinical Medical Sciences approved the study protocol (Approval no. 13005), and experiments were performed in accordance with the NIH Guiding Principles for the Care and Use of Laboratory Animals.

Mice were monitored carefully daily, including signs of illness, pain, or moribundity. All mice were anaesthetized followed by euthanasia by cervical dislocation. Humane endpoints of mice were also set up when mice exhibited weight loss of 15%, or being unable to obtain food or water, or moribund state. No mice showed signs of pain or moribund state in this study, so all mice were humane euthanasia after 12 weeks of treatment with or without TSF.

### Urinary albumin excretion and renal tissue pathology

Mice were kept in metabolic cages (Fengshi Inc., Suzhou, JS, China) to collect a 24-hour urinary a 4-week interval to measure urine volume. Levels of urinary albumin were analyzed using enzyme linked immunosorbent assay (ELISA) Quantitation Set (Bethyl Laboratories Inc., Montgomery, TX) according to the manufacturer’s instructions. Kidney tissues were fixed, embedded in paraffin and stained with periodic acid-Schiff (PAS), which were examined by light microscopy. Glomerulosclerosis was defined as the percentage of ECM deposition and mesangial expansion and evaluated at 400× power for 20 cortical fields. The mesangial matrix was scored as follows: 1, < 10%; 2, 10–25%; 3, 26–50%, 4, 51–75%, 5, 76–95%, 6, > 95%. Immunohistochemistry was conducted using the paraffin sections of mice kidney. The primary antibody against PLZF (sc-22839; Santa Cruz) was applied. The immunohistochemistry was analyzed using GTVisionTM Ⅲ Detection System/Mo&Rb (GK500705, GeneTech, Shanghai, China) according to the manufacturer’s instructions. Deposition of PLZF in mice kidney was measured using the quantitative Image Analysis System (Image-Pro Plus 6.0, Media Cybernetics, Warrendale, PA, USA). 10 random and discontiguous fields were selected and positive staining patterns were identified under 200× power, and the IOD (integrated option density) values in the examined field were measured. Data were showed as the means ± SEM.

### RNA extraction and real-time PCR analysis

Renal cortical tissues were carefully collected and frozen at -80°C. Total RNA was extracted from renal cortical tissues and NRK52E cells using TRIZOL (Invitrogen, Carlsbad, CA, USA), and then reverse transcription was conducted by the use of a kit (Thermo Scientific, Waltham, MA, USA). The manufacturer’s instructions (ABI system, USA) for real-time PCR were used to determine the levels of expression of the target genes. The primer sequences used in the present study are listed in Table 1.

**Table 1:** Primers used for quantitative real-time PCR.

Gene	Sense	Primer sequence 5‘ -3'	Product size (bp)
***Abcb1b***	Forward	5’-GAAGCCAGTATTCTGCCAAGC-3’	241
	Reverse	5’-ACCAGCCTATCTCCTGATTCATTAT-3’	
***Pgm5***	Forward	5’-CATCCAGAGTGTGCTGTCGTC-3’	196
	Reverse	5’-CCTGATAATGCAGGAGACTGC-3’	
***PLZF***	Forward	5’-CCCAGTTCTCAAAGGAGGATG-3’	88
	Reverse	5’-TTCCCACACAGCAGACAGAAG-3’	
***β-actin***	Forward	5’-ACCCTAAGGCCAACCGTGAAAAG-3’	239
	Reverse	5’-CATGAGGTAGTCTGTCAGGT-3’	

### Microarrays

Microarray was conducted in Capital Bio Corporation (Beijing, China) using Affymetrix GeneChip, which was authorized by Affymetrix, Inc. (Santa Clara, CA, USA). In microarray experiments, 100ng of total RNA was used for cDNA synthesis. Biotin-tagged cRNA was produced using the GeneChip IVT Labeling Kit (Affymetrix). Subsequently, 15μg fragmented cRNA, Control Oligo B2 and eukaryotic hybridization controls was hybridized to Affymetrix Mouse Genome 430 2.0 Array at 45°C for 16 h in Affymetrix GeneChip Hybridization Oven 640 (Affymetrix, Santa Clara, CA). The GeneChip arrays were then washed and stained with streptavidin phycoerythrinonan with Affymetrix GeneChip FluidicsStation 450, followed by scanning with Affymetrix GeneChip Scanner 3000 7G. Data were analyzed using Affymetrix Expression Console and Transcriptome Analysis Console software. Gene array was run in triplicate, and the significance for each gene was determined by one-way ANOVA. 1.5-fold change value was defined as genes with different expression.

### Cell culture and grouping

Rat renal proximal tubular epithelial cell lines (NRK52E), which were purchased from ATCC (Rockville, MD, USA), were utilized in the *in vitro* experiments. The cells were cultured in Dulbecco’s modified Eagle’s medium (DMEM low glucose; hyclone; USA) supplemented with 5% FBS (Gibco, USA). A humidified incubator with 5% CO_2_ at 37°C was used to culture the NRK52E cells. The cells were divided into the following groups: NG, the normal-glucose group, which contained 5.5 mmol/L glucose; HG, the high-glucose group, which consisted of 30 mmol/L glucose (Sigma). HG+TSF250 pertained to the 250 μg/mL TSF intervention group, which contained 30 mmol/L glucose + 250 μg/mL TSF. HG+TSF500 represented the 500 μg/mL TSF intervention group, which comprised 30 mmol/L glucose + 500 μg/mL TSF. HG+TSF750 was the 750 μg/mL TSF intervention group, which consisted of 30 mmol/L glucose + 750 μg/mL TSF.

### Detection of cytotoxicity and cell proliferation using a CCK8 assay

The NRK52E cells were plated into 96-well plates at a density of 1.5 × 10^3^ cells/well. After culturing for 24 h, the culture medium was replaced with DMEM supplemented with glucose or TSF. After 24 h, 48 h, or 72 h of culture, 10 μL of a cell counting solution (CCK8, Dojindo, Japan) was added to each well. The cells were then placed in an incubator for 1 h, and the optical density (OD) of each well at a wavelength of 450 nm was measured using a microplate reader and used in calculating the rates of cell proliferation and cell survival. For the cytotoxicity assay, TSF concentrations of 100 μg/mL, 250 μg/mL, 500 μg/mL, 750 μg/mL, and 1,000 μg/mL were used, and cell survival rate was assessed by CCK8 according to the manufacturer’s instructions.

Calculation formula:

Cell survival rate = [(As-Ab) / (Ac-Ab)] × 100%

As: Experiment wells (culture medium containing cells, CCK8 and TSF);

Ac: Control wells (culture medium containing cells and CCK8, without TSF);

Ab: Blank wells (culture medium containing CCK8, without cells and TSF).

### Transmission electron microscopy

The cells were digested, centrifuged, and collected after 72 h of culture, and after twice washes with cold PBS, the cells were fixed in 5% (w/v) glutaraldehyde. The cells were then post-fixed in 1% (w/v) osmium tetroxide, dehydrated by the concentration gradient of ethanol (50%, 70%, 80%, 90% and 95%), and embedded by Epon812. Then, sections were cut at 0.12 μm thickness and stained using 1% (w/v) uranyl acetate and 0.2% (w/v) lead citrate. The autophagosomes were observed by transmission electron microscopy (JEOL-100CXII, JEOL, Japan). 10 fields (8000×) from each group of cells were randomly selected, and the number of intracellular autophagosomes was counted.

### Plasmid construction and cell transfction

The coding sequence of the *ZBTB16/PLZF* gene of the NRK52E cells was amplified using RNA as template, which included BamHI and EcoRI restriction sites, respectively. The primers for real-time PCR amplification were as follows: forward primer, 5'-CGCGGATCCATGGATCTGACAAAAATGGGC-3', and reverse primer, 5'-CCGGAATTCCACATAGCACAGGTAGAGGTA-3'. Double-stranded siRNAs for *PLZF* gene were synthesized and purified using high-performance liquid chromatography (GenePharma, Shanghai, China). The PCR amplification products were sub-cloned into a pcDNA3.1A vector (pcDNA3.1A-PLZF), and the sequence of construct was validated by Sanger sequencing. The siRNA sequences targeting the *PLZF* gene (siPLZF) were 5'-GGGUCGAGCUUCCUGAUAATT-3' (forward) and 5'-UUAUCAGGAAGCUCGACCCTT-3' (reverse), and 5'-UUCUCCGAACGUGUCACGUTT-3' (forward) and 5'-ACGUGACACGUUCGGAGAATT-3' (reverse) for the negative control (NC).

The NRK52E cells were seeded into 6-well plates at a density of 8 × 10^4^ cells per well the day before transfection. Approximately 1 μg or 2 μg of pcDNA3.1A and pcDNA3.1A-PLZF were transiently transfected into the NRK52E cells using Lipofectamine 2000 (Invitrogen, Carlsbad, CA, USA), and 20 μM NC and siPLZF were transfected into cells that were cultured with 5.5 mM or 30 mM glucose, respectively. After 48 h of culture, the cells were harvested for further analysis.

### Western blot analysis

Equal amounts (20 μg) of nuclear and cytoplasmic extracts were used in 12% SDS-PAGE analysis, which were then transferred to a polyvinylidene difluoride membrane and then blocked in 5% nonfat milk in Tris-buffered saline with Tween-20 (TBST, 0.1%) for 1 h at room temperature, and then immunoblotted with the corresponding primary antibodies, which included rabbit PLZF (sc-22839; Santa Cruz), rabbit LC3 II (L7543; Sigma), rabbit p62/SQSTM1 (PM045; MBL), rabbit collagen III (ab7778; Abcam), and mouse β-actin (sc-70319; Santa Cruz), and incubated overnight at 4°C. After washing with TBST, the membranes were incubated with the appropriate secondary antibodies for 1 h at room temperature and then detected using a ChemiDoc XRS system (Bio-Rad, CA, USA). Finally, Image J (NIH, USA) was used to quantify the protein bands.

### Statistical analyses

All analyses were performed using SPSS 20.0. The quantitative data were expressed as the mean ± SEM. Independent-samples t-test or ANOVA was applied for the statistical analysis. Pair-wise comparisons were performed using the t-test. P<0.05 was accepted as significance.

## Results

### TSF alleviated proteinuria and histological damage in kidneys of db/db mice with T2DM

**Fig 1:**
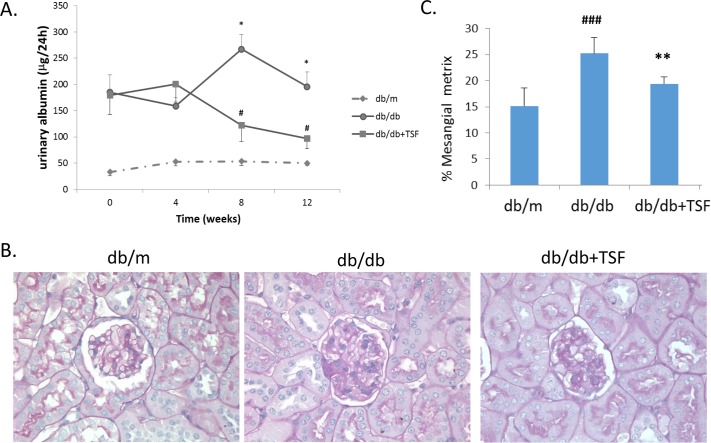
TSF alleviated urine albumin levels and glomerular extracellular matrix deposition in db/db mice. (A) Administration of TSF effectively diminished urine albumin in db/db mice. (B) and (C) Treatment with TSF decreased glomerular mesangial matrix deposition in db/db mice. The data were expressed as the mean ± SEM. #P<0.05, ###P<0.001 vs. db/m group; *P<0.05, **P<0.01 vs. db/db group.

24-hour urinary protein (24h UP) was observed in the db/m, db/db and db/db mice with TSF treatment at the first week (termed week 0), fourth week, eighth week, and 12th week. Compared to the db/m mice, 24h UP in the db/db mice markedly increased on week 0, which was then maintained for the entire study period (a total of 12 weeks). Moreover, TSF significantly attenuated proteinuria in the db/db mice from the eighth to 12th week (Fig 1A).

Histologically, mesangial matrix expansion and extracellular matrix deposition were observed in the kidneys of the db/db mice, and these histological injuries were significantly ameliorated in the db/db mice that were treated with TSF (Fig 1B and 1C).

### Analysis and validation gene expression profiling in db/db mice with T2DM

**Fig 2:**
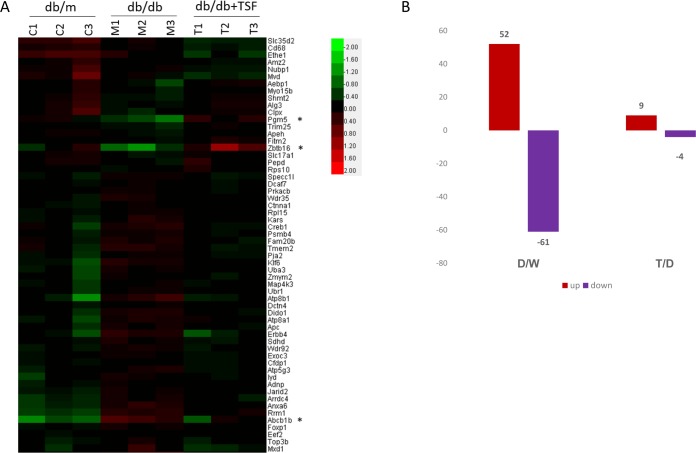
Gene expression microarray profiling. (A) Heat map of microarray gene expression profiles of kidney tissue samples of db/m (n = 3), db/db (n = 3), and db/db (n = 3) mice treated with TSF; (B) The number of genes that were differentially expressed in the 3 groups; D, db/db mice; M, db/m mice; and T, db/db mice+TSF.

To identify the pharmacologic target of TSF in renal injuries in T2DM, mRNA profiling of the kidneys of db/m (n = 3), db/db (n = 3), and db/db mice subjected to TSF treatment (n = 3) was performed using Affymetrix gene expression microarray analysis. The levels of gene expression significantly differed among the three groups of mice. A total of 52 genes were upregulated, whereas 61 genes were downregulated in the kidneys of db/db mice compared to that in the db/m mice. Compared to the db/db mice, 9 genes were upregulated and 4 genes were downregulated in the db/db mice treated with TSF (Fig 2), of which 3 genes were differentially expressed among the 3 groups: *Pgm5*, *Zbtb16/PLZF*, and *Abcb1b*. The fold-change and P-values derived from the microarray analysis were presented in Table 2.

**Table 2:** *Pgm5*, *Zbtb16/PLZF*, and *Abcb1b* expression microarray profiling.

Gene	Fold change		P-Value	
	D/M	T/D	D/M	T/D
***Pgm5***	0.626	1.760	0.014	0.011
***Zbtb16/PLZF***	0.591	2.74	0.076	0.077
***Abcb1b***	2.411	0.641	0.003	0.058

D, db/db mice; M, db/m mice; and T, db/db mice+TSF.

**Fig 3:**
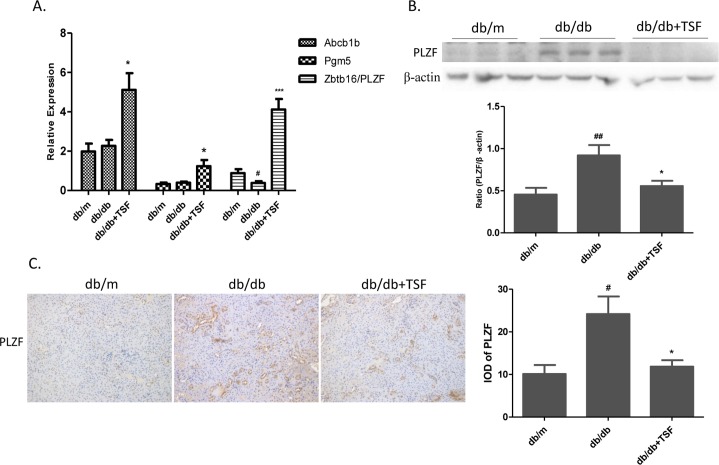
Validation of the expression levels of 3 genes. (A) The relative expression levels of 3 genes were normalized to that of β-actin. (B) Western blot analysis was used to validate the expression level of PLZF in the 3 groups. (C) Immunohistochemistry and semi-quantitative analysis for PLZF. The data were expressed as the mean ± SEM. #P<0.05, ###P<0.001 vs. db/m group; *P<0.05, **P<0.01 vs. db/db group.

To confirm the findings of gene expression profiling microarray analysis, real-time PCR was performed to measure mRNA expression of the 3 genes (Fig 3A). No significant differences in the levels of *Pgm5* and *Abcb1b* expression between the db/m and db/db mice were observed, and only *PLZF* was downregulated in the kidneys of the db/db mice compared to that in the db/m mice, which was then reversed by TSF treatment. To further confirm the expression levels of PLZF, western bolt analysis and immunohistochemistry analysis of the kidneys of the db/m (n = 9), db/db (n = 9), and db/db (n = 9) mice subjected to TSF treatment were conducted. However, we observed the opposite results, in which PLZF was upregulated in the db/db mice compared to that in the db/m mice, whereas the downregulation of PLZF was detected in TSF-treated db/db mice compared to that in the db/db mice. We also found that PLZF protein mainly located in mouse renal tubular epithelial cells (Fig 3B and 3C). These findings suggested that epigenetic regulation or post-transcriptional modifications might be involved in the process of diabetogenesis.

### TSF inhibited PLZF and collagen III accumulation, and promoted autophagy in the kidneys of T2DM mice

**Fig 4:**
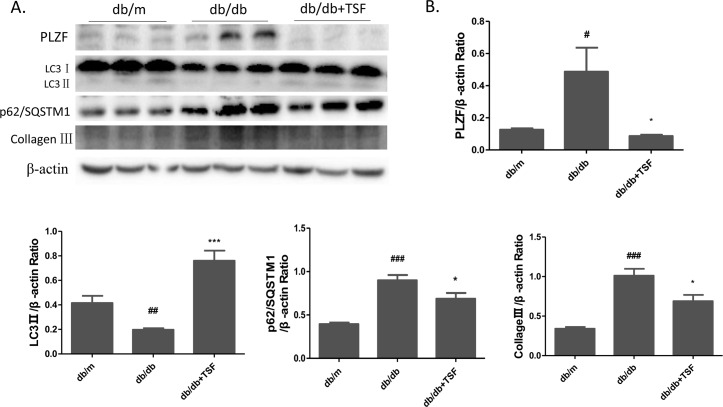
TSF treatment increased renal autophagy levels and decreased collagen III accumulation in db/db mice. (A) and (B) Western blot was performed to assess the abundance of PLZF, LC3 II, p62/SQSTMI, and collagen III in the mouse kidneys. The data were expressed as the mean ± SEM. #P<0.05, ## P<0.01, ### P<0.001 db/db group vs. db/m group; *P<0.05, ***P<0.001 TSF-treated group vs. db/db group.

One of the main symptoms of DN was the accumulation of collagen in the glomerular and tubular. Recent studies have suggested that autophagy involved the negative regulation of PLZF, which mediated the ubiquitination and proteasomal degradation of the autophagic protein Atg14L [17]. Furthermore, defects in autophagic degradation contributed to the accumulation of collagen in diabetic renal injuries [11]. To explore whether the underlying mechanism of PLZF in autophagy and collagen deposition participated in the pathogenesis of DN, western blot was conducted to verify expression level of autophagy marker proteins and collagen III in mouse kidneys. In the db/db mice, the LC3 II expression was significantly downregulated, whereas that of p62/SQSTMI was significantly upregulated compared to that in the db/m mice, thereby indicating autophagy inhibition. After TSF treatment, LC3 II expression was significantly upregulated whereas that of p62/SQSTMI was significantly downregulated in TSF-treated mice compared to that in the db/db mice. Furthermore, we also observed a higher level of collagen III expression in the kidneys of db/db mice than that in db/m mice, which was reversed by TSF treatment (Fig 4A and 4B).

### TSF restored cell proliferation in HG-induced NRK52E cells

**Fig 5:**
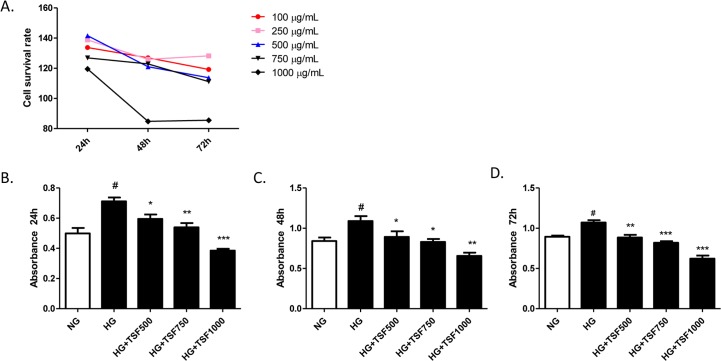
TSF treatment on HG-induced NRK52E cells resulted in a significant reduction in cell proliferation rate. (A) TSF cytotoxicity was reflected by the cell survival rate, which was assessed using the CCK8 assay. (B) The CCK8 assay was also used to determine the cell proliferation rate of NRK52E cells cultured for 24 h, 48 h, and 72 h. The data were expressed as the mean ± SEM. #P<0.05, HG group vs. NG group; *P<0.05, ***P<0.001 HG+TSF group vs. HG group.

Cell proliferation in renal proximal tubular cells has been considered to play an important role in the progression of DN. To determine whether TSF affected cell proliferation, CCK8 assays were performed to investigate the cytotoxicity of TSF and its effect on the proliferation of NRK52E cells. In the cytotoxicity assay, the NRK52E cells were treated with TSF at various concentrations, ranging from 100 μg/mL to 1000 μg/mL. The application of TSF at concentrations of 100 μg/mL, 250 μg/mL, 500 μg/mL, and 750 μg/mL did not result in any significant changes in the survival rate of NRK52E cells. However, a significant decrease in the rate of cell survival was observed in cells treated with 1,000 μg/mL TSF, indicating that this concentration was cytotoxic to NRK52E cells. Based on these findings, TSF concentrations <750 μg/mL were used in the subsequent experiments (Fig 5A).

To further confirm the effect of TSF on cell proliferation, NRK52E cells were treated with high glucose (HG) or HG combined with TSF (HG+TSF) at concentrations of 500 μg/mL, 750 μg/mL, and 1,000 μg/mL. After treatment for 24 h, 48 h, and 72 h, a significant increase in cell proliferation was observed in the HG group compared to that in the NG group. The cells that were exposed to HG combined with 500 μg/mL or 750 μg/mL TSF for 24 h, 48 h, and 72 h showed a lower cell proliferation rate than that in the HG group. In addition, possibly due to the cytotoxic effects of TSF, HG-induced cells treated with 1,000 μg/mL TSF always showed a significantly reduced level of cell proliferation (Fig 5B–5D).

### TSF reversed HG-induced PLZF and collagen accumulation, and autophagy inhibition in NRK52E cells

**Fig 6:**
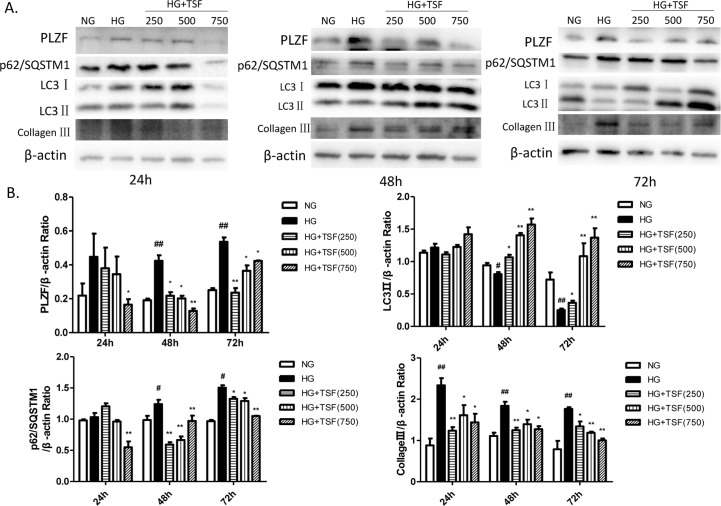
TSF inhibited PLZF upregulation and collagen III accumulation, and restored autophagy levels in NRK52E cells exposed to high levels of glucose. (A) Western blot analysis of PLZF, LC3 II, p62/SQSTMI, and collagen III expression in NRK52E cells cultured for 24 h, 48 h, and 72 h. (B) The intensities of the each protein band were quantified. The data were expressed as the mean ± SEM. #P<0.05, ## P<0.01, HG group vs. NG group; *P<0.05, **P<0.01 HG+TSF group vs. HG group.

Based on the results of the animal experiments, we were then prompted to examine the effect of HG and TSF on the expression of PLZF, autophagy markers (LC3 II and p62/SQSTMI), and collagen III in NRK52E cells by western blot analysis. No significant differences in PLZF and autophagy proteins expression among the 3 groups after 24 h of treatment were observed. Compared to the NG group, PLZF and p62/SQSTMI were significantly upregulated, whereas LC3 II was significantly downregulated in the HG group cultured for 48 h and 72 h. Compared to the HG group, PLZF and p62/SQSTMI expression were significantly downregulated and LC3 II was significantly upregulated in cells exposed to different concentrations of TSF for 48 h and 72 h. Furthermore, collagen III was significantly upregulated in the HG group after 24 h, 48 h, and 72 h of treatment. Compared to the HG group, collagen III was significantly downregulated after treatment with different TSF concentrations for 24 h, 48 h, and 72 h. Together, these data indicated that TSF could reverse HG-induced PLZF, p62/SQSTMI, and collagen III upregulation, as well as LC3 II downregulation in NRK52E cells (Fig 6A and 6B).

**Fig 7:**
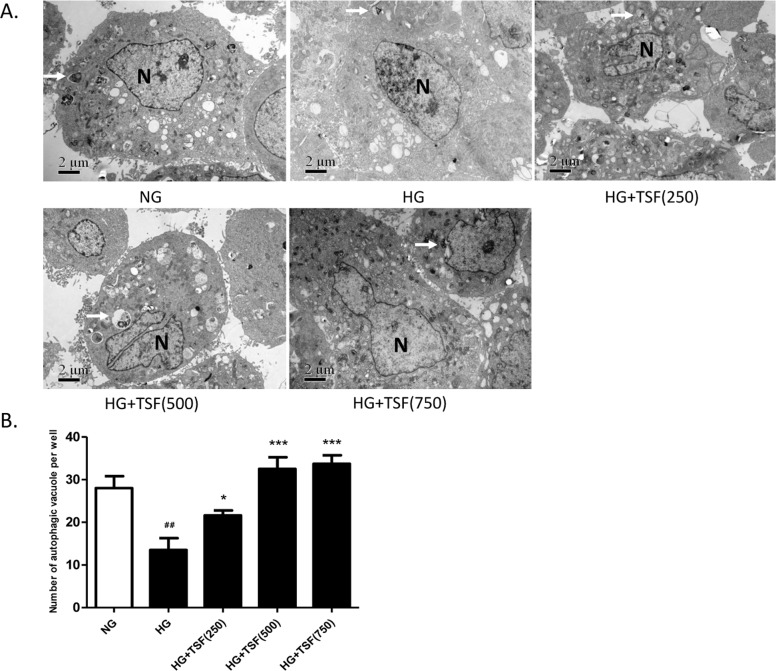
TSF increased the number of autophagic vacuoles in NRK52E cells. (A) Transmission electron microscopy (8000x) micrograph. The arrows indicated autophagosomes and “N” represents nucleus. Autophagic vacuoles with typical double-layer membrane structures containing undigested organelle remnants were indicated with arrows. Bar = 2 μm. (B) The number of autophagosomes was counted in 10 randomly selected fields. ##, P<0.01 vs. NG; *, P<0.05 vs. HG; ***, P< 0.001 vs. HG.

The autophagosomes in NRK52E cells cultured for 72 h from each group were examined through transmission electron microscopy. The number of autophagosomes (8000x) was counted in 10 fields for each group. Compared with the NG group, the number of autophagosomes in cells of the HG group was significantly decreased (Fig 7A and 7B). The number of autophagosomes in NRK52E cells in HG group with 250 μg/mL TSF was significantly increased compared with the HG group, and the number of autophagosomes was further increased in cells with 500 μg/mL or 750 μg/mL TSF (Fig 7A and 7B).

### PLZF aggravated collagen III accumulation by negatively regulating autophagy in NRK52E cells

**Fig 8:**
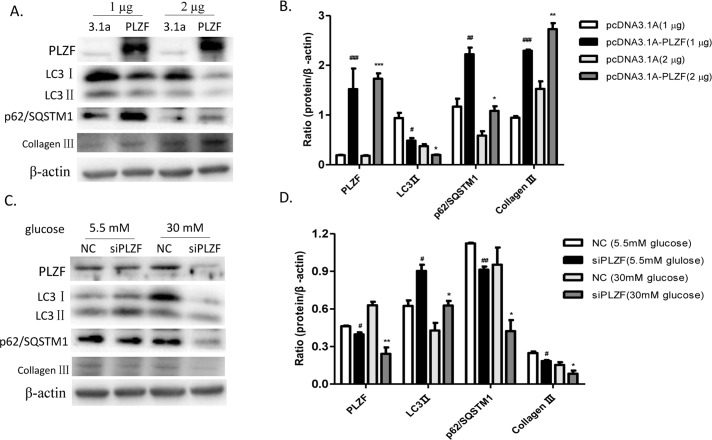
PLZF reduced cellular autophagy and promoted collagen III accumulation in NRK52E cells. (A) and (B) The levels of PLZF, LC3 II, p62/SQSTMI, and collagen III expression in NRK52E cells. (C) and (D) The levels of PLZF, LC3 II, p62/SQSTMI, and collagen III expression were assessed in PLZF-knockout NRK52E cells. Data were expressed as the mean ± SEM. #P<0.05, ## P<0.01, ### P<0.001 HG group *vs*. NG group; *P<0.05, **P<0.01, ***P<0.001 HG+TSF group *vs*. HG group.

To determine whether PLZF contributed to the expression of autophagy markers and collagen III in NRK52E cells, we constructed PLZF recombinant plasmids and siPLZF. To confirm the effect of PLZF overexpression on autophagy proteins and collagen III expression, we transfected the NRK52E cells with a pcDNA3.1A empty vector and pcDNA3.1A-PLZF recombinant plasmids at different concentrations, respectively. Cells with pcDNA3.1A-PLZF showed an upregulation of PLZF, p62/SQSTMI and collagen III, and a downregulation of LC3 II compared to that in cells transfected with pcDNA3.1A vectors. However, no significant dose-dependent changes in protein expression levels were observed in the cells transfected with the pcDNA3.1A-PLZF plasmids (Fig 8A and 8B). In addition, NC and siPLZF were transfected into the NRK52E cells, which were respectively cultured in media supplemented with 5.5 mM and 30 mM glucose. Using 5.5 mM glucose, a slight decrease in the expression of PLZF was observed, which apparently resulted in the upregulation of LC3 II and the downregulation of p62/SQSTMI and collagen III. Moreover, incubation in 30 mM glucose apparently enhanced the downregulation of PLZF in NRK52E cells that were transfected with siPLZF compared to that in the NC, which showed higher autophagy levels and collagen III expression (Fig 8C and 8D). Together, these findings indicated that PLZF negatively regulated cellular autophagy and positively regulated collagen III expression in NRK52E cells.

## Discussion

Previous studies have shown that the Chinese herbal granule TSF decreased proteinuria and improved eGFR in diabetic kidney disease patients, although little was known on the underlying mechanisms. In the present study, we demonstrated that PLZF might be potentially utilized as a therapeutic target of TSF in the treatment of diabetic kidney injuries, which possibly influenced autophagy and collagen deposition.

Earlier studies have conducted microarray expression profiling to explore the molecular targets of Chinese herbal granules in disease therapy [18, 19]. In the present study, we successfully validated the renoprotective effects of TSF in a db/db T2DM mouse model and determined that TSF targeted PLZF expression in DN. Compared to db/m mice, proteinuria and mesangial matrix expansion significantly increased in db/db mice, which were inhibited after 8 weeks of treatment with TSF. By analyzing and validating the results of microarray expression profiling, we have demonstrated that PLZF might be the target of TSF. The protein expression of which was upregulated in db/db mice and subsequently downregulated after TSF-treatment. However, the relative mRNA expression level of PLZF decreased in db/db mice compared to that in control mice, and microarray and real-time PCR analyses showed that this increased after TSF treatment. The inconsistence of mRNA and protein level of PLZF was possibly due to epigenetic regulation or post-transcriptional modifications.

Previous microarray studies listed in the GEO database that involved peripheral blood mononuclear cells (PBMCs) from type 1 diabetes patients have indicated that PLZF was a candidate gene for type 1 diabetes [20]. However, these reports did not validate the levels of PLZF protein expression. To date, little has been known about PLZF expression in DN. Recent research evidence has demonstrated that PLZF was an important member of the ZBTB16/PLZF-Cullin3-Roc1 E3 ubiquitin ligase complex, and its expression was negatively correlated with ATG14L, a key autophagy protein, thereby indicating that PLZF was a negative regulator of autophagy [17]. Furthermore, hyperglycemia has been shown to impair cellular autophagy in T2DM animal models, which was also coupled with the upregulation of p62/SQSTM1 [21]. Thus, PLZF might be involved in the mechanism of DN via the negative regulation of autophagy, and might thus be used as a molecular target of TSF for the treatment of DN. The present study showed that db/db mice had lower renal autophagy and LC3 II and higher p62/SQSTMI protein expression levels compared to that in db/m mice, and TSF significantly inhibited cellular autophagy in the kidneys of db/db mice. *In vitro* experimentation also showed that TSF increased PLZF expression and decreased HG-induced autophagy in NRK52E cells. In addition, PLZF-overexpressing NRK52E cells displayed a lower level of autophagy, whereas PLZF-knockdown cells showed a higher level of autophagy. These findings implicated PLZF played a part in cellular autophagy-mediated diabetic renal injuries and might be used as the target protein of TSF during DN treatment. However, further investigations on the role of PLZF in diabetic renal injuries in relation to autophagy were warranted.

ECM expansion in the mesangium and renal tubular epithelial cells were hallmark features of T2DM DN. Previous reports have shown that ECM production was negatively regulated by autophagy, which promoted the degradation of intracellular type 1 collagen (collagen I) and type 3 collagen (collagen III) in renal tubular epithelial cells [22, 23]. Moreover, earlier studies have suggested promoting AGE clearance had renoprotective effects, which were formed by the crosslinking of glucose with ECM proteins [24]. Consistent with our expectations, collagen III expression levels were elevated in the kidneys of db/db mice compared to that in db/m mice in this study, and were also increased in the HG group NRK52E cells compared to that in the NG group. TSF intervention markedly decreased collagen III protein expression in db/db mice and in HG-induced NRK52E cells. Furthermore, PLZF-overexpressing NRK52E cells showed significantly higher collagen III levels than that in control cells, and PLZF knockdown cells had lower levels collagen III expression. Thus, TSF could effectively inhibit collagen III accumulation in diabetic kidney injuries, in which PLZF might play a key role.

In the current study, cytotoxicity and cell proliferation assays in NRK52E cells were also conducted using a CCK8 kit. TSF at a concentration of 750 μg/mL showed no apparent significant cytotoxic effects. Cells cultured with HG displayed a higher proliferation rate compared to that in the NG group, which was significantly reversed by TSF treatment. However, PLZF has been proven to be an anti-oncogene that negatively regulated cell proliferation [4]. Here, abnormal cell proliferation was observed after HG treatment for 24 h; while abnormal levels of PLZF protein expression were observed after 48 h. These results illustrated that cell proliferation occurs prior to abnormal PLZF expression. TSF could therefore inhibit the proliferation of HG-induced NRK52E cells, its underlying mechanism might not be related to PLZF expression. Our *in vitro* experiments showed that although no abnormal PLZF expression and autophagy levels were observed in NRK52E cells cultured with HG for 24 h, we observed collagen III accumulation, which was restored by TSF treatment. These findings suggest that collagen III accumulation might be driven by cell proliferation, therefore, cell proliferation might be another underlying mechanism in the treatment of DN using TSF. Further research on the mechanisms involved in the effect of TSF on renal tubular cell proliferation are thus warranted.

## Conclusions

The present study demonstrated that PLZF might be the therapeutic target of TSF in the treatment of DN. Furthermore, we determined that HG upregulated PLZF, which in turn inhibited autophagy, thereby causing a reduction in autophagy-mediated degradation of collagen III, ultimately resulting in ECM deposition in diabetic renal tubular cells, and TSF improved collagen deposition by downregulating PLZF expression.
